# The Polyphosphate Kinase of Escherichia coli Is Required for Full Production of the Genotoxin Colibactin

**DOI:** 10.1128/mSphere.01195-20

**Published:** 2020-12-16

**Authors:** Min Tang-Fichaux, Camille V. Chagneau, Nadège Bossuet-Greif, Jean-Philippe Nougayrède, Éric Oswald, Priscilla Branchu

**Affiliations:** a IRSD, Université de Toulouse, INSERM, INRAE, ENVT, UPS, Toulouse, France; b CHU Toulouse, Service de Bactériologie-Hygiène, Toulouse, France; University of Kentucky

**Keywords:** *pks*, genotoxicity, PPK, mesalamine, colibactin

## Abstract

Colibactin induces DNA damage in mammalian cells and has been linked to the virulence of Escherichia coli and the promotion of colorectal cancer (CRC). By looking for mutants attenuated in the promoter activity of *clbB* encoding one of the key enzymes for the production of colibactin, we found that a mutant of the gene coding for the polyphosphate kinase (PPK) produced less colibactin than the parental strain. We observed this phenotype in different strains ranging from pathogens responsible for meningitis, urinary tract infection, or mouse colon carcinogenesis to the probiotic Nissle 1917. We confirmed the role of PPK by using an inhibitor of PPK enzymatic activity, mesalamine (also known as 5-aminosalicylic acid). Interestingly, mesalamine has a local anti-inflammatory effect on the epithelial cells of the colon and is used to treat inflammatory bowel disease (IBD). Upon treatment with mesalamine, a decreased genotoxicity of colibactin-producing E. coli was observed both on epithelial cells and directly on purified DNA. This demonstrates the direct effect of mesalamine on bacteria independently from its anti-inflammatory effect on eukaryotic cells. Our results suggest that the mechanisms of action of mesalamine in treating IBD and preventing CRC could also lie in the inhibition of colibactin production. All in all, we demonstrate that PPK is required for the promoter activity of *clbB* and the production of colibactin, which suggests that PPK is a promising target for the development of anticolibactin and antivirulence strategies.

**IMPORTANCE** Colibactin-producing E. coli induces DNA damage in eukaryotic cells and promotes tumor formation in mouse models of intestinal inflammation. Recent studies have provided strong evidence supporting the causative role of colibactin in human colorectal cancer (CRC) progression. Therefore, it is important to understand the regulation of the production of this genotoxin. Here, we demonstrate that polyphosphate kinase (PPK) is required for the promoter activity of *clbB* and the production of colibactin. Interestingly, PPK is a multifunctional player in bacterial virulence and stress responses and has been proposed as a new target for developing antimicrobial medicine. We observed inhibition of colibactin production by using a previously identified PPK inhibitor (i.e., mesalamine, an anti-inflammatory drug commonly prescribed for inflammatory bowel diseases). These data brought us a new perspective on the regulatory network of colibactin production and provided us a clue for the development of anticolibactin strategies for CRC treatment/prophylaxis.

## Abstract (precis)

Colibactin-producing E. coli induces DNA damage in eukaryotic cells and promotes tumor formation in mouse models of intestinal inflammation. Recent studies have provided strong evidence supporting the causative role of colibactin in human colorectal cancer (CRC) progression.

## INTRODUCTION

Colibactin is a natural genotoxic compound produced primarily by Escherichia coli carrying the 54-kb *pks* genomic island (1). The *pks* island, harboring 19 genes (*clbA* to *clbS*), encodes enzymes responsible for the synthesis of colibactin. Nonribosomal peptide synthases (NRPSs; i.e., ClbN, ClbH, and ClbJ), polyketide synthases (PKSs; i.e., ClbC, ClbI, and ClbO), and hybrid NRPS-PKSs (i.e., ClbB and ClbK) constitute an assembly line which is activated by phosphopantetheinyl transferase ClbA (2). Following activation by ClbA, the initiating NRPS ClbN uses asparagine as a substrate to generate the prodrug motif *N*-myristoyl-d-Asn (C_14_AsnOH) (3). Then, ClbB accepts C_14_AsnOH and constructs the amide bond cleavable by the periplasmic membrane-bound peptidase ClbP (3). With continuous actions of other enzymes on the assembly line, precolibactin is synthesized in the cytoplasm and then exported to the periplasm, where precolibactin is cleaved by ClbP to release active colibactin and the prodrug motif C_14_AsnOH (3).

The warheads of colibactin alkylate DNA on two adenine residues of opposite strands of DNA, which induces a DNA interstrand cross-link (ICL) and ultimately a DNA double-strand break (DSB) (4, 5). These types of DNA damage in eukaryotic cells activate DNA repair pathways, resulting in histone H2AX phosphorylation (producing γH2AX) and senescence (1, 4, 6). Colibactin has been linked to bacterial virulence (7, 8) and microbial diversity (9). *In vivo*, colibactin-producing E. coli has been shown to cause DNA damage (10, 11) and tumor formation (12–15) in mouse models of intestinal inflammation. Importantly, a high abundance of colibactin-producing E. coli has been found in inflammatory bowel disease (IBD) and colorectal cancer (CRC) patients (12, 15, 16). Furthermore, recent studies have revealed colibactin DNA damage signatures that directly indicate the mutational impact in CRC (4, 6).

Given the role of colibactin in bacterial virulence and tumorigenesis, it is important to understand the regulation of its production, to provide clues for the development of anticolibactin strategies. It was recently reported that ClbR is an (auto)transcriptional activator of the *clbB* gene (17). In addition, the two master regulators of bacterial iron homeostasis Fur (ferric uptake regulator) and the small regulatory noncoding RNA RyhB regulate the transcription of *clbA* (18, 19). *In vivo* studies showed that the expression of *pks* genes was upregulated in human urine (20) and enriched in intestinal inflammation and CRC development (21–23).

In this work, we used a random mutagenesis strategy to find regulators involved in colibactin production. We determined that a mutant of the gene *ppk* encoding polyphosphate kinase (PPK) has a lower *clbB* promoter (P*clbB*) activity than the wild type (WT). PPK catalyzes the reversible conversion of the terminal (γ) phosphate of ATP to long chains of inorganic polyphosphate (polyP; ca. 750 residues), which has been found to be involved in bacterial virulence and stress responses (24). In this work, we found that PPK played a positive role for P*clbB* activity and colibactin production. As mesalamine (also known as 5-aminosalicylic acid) is an inhibitor of PPK enzymatic activity (25), we tested and confirmed that this commonly prescribed drug is capable of inhibiting P*clbB* activity and colibactin biosynthesis.

## RESULTS

### Identification of PPK as an enhancer of P*clbB* activity.

**FIG 1:**
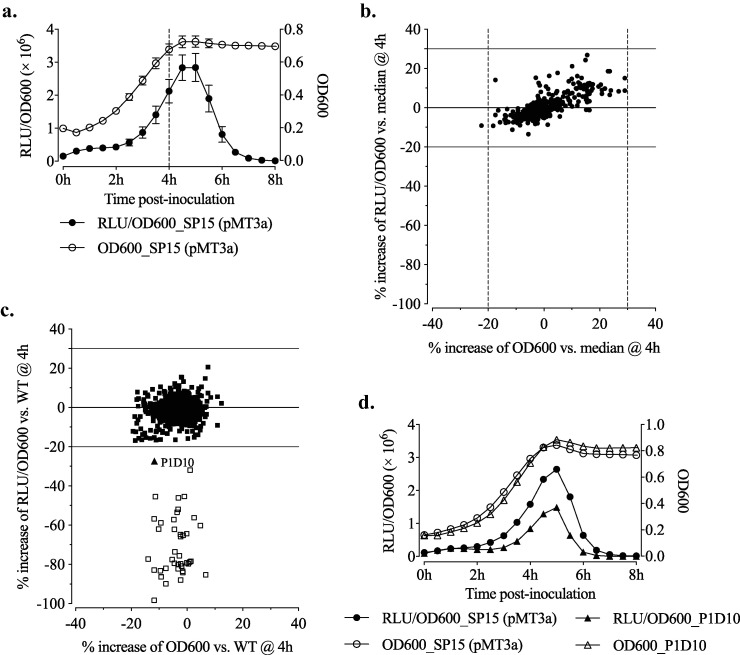
Identification of a mutant of *ppk* having attenuated P*clbB* activity. (a) Time course of P*clbB* activity and growth of SP15(pMT3a) in DMEM-HEPES at 37°C. RLUs were standardized by OD_600_ (RLU/OD_600_) as the readout for P*clbB* activity. Values are means ± SEMs (*n* = 6 biological replicates). (b) At 4 h, RLU/OD_600_ and OD_600_ of 300 isolates of SP15(pMT3a) were determined. Each black circle represents the increase of RLU/OD_600_ (*y* axis) and OD_600_ (*x* axis) versus the median of 300 isolates. (c) Screening of P*clbB* activities in 806 Tn mutants of SP15(pMT3a) at 4 h in DMEM-HEPES at 37°C. The increase of RLU/OD_600_ or OD_600_ versus the WT are shown. Black squares represent 765 mutants having a variation of increased RLU/OD_600_ or OD_600_ between −20% and 30%. The black triangle represents the mutant P1D10 having −27% increase of RLU/OD_600_ versus WT. White squares represent 40 mutants having less than a −20% increase of RLU/OD_600_ versus that of the WT. (d) Time course P*clbB* activity and growth of P1D10 and the WT [SP15 (pMT3)] in DMEM-HEPES at 37°C. Data are means from two independent experiments.

On the assembly line of colibactin, ClbB is the enzyme that accepts the prodrug motif C_14_AsnOH and constructs the amide bond cleavable by ClbP for releasing active colibactin (3). In order to investigate the regulation of colibactin production, we constructed a transcriptional fusion expressing *luxCDABE* (*lux*) under the control of P*clbB*, resulting in plasmid pMT3a (Table 1; see also Fig. S1a in the supplemental material). This plasmid was transformed into E. coli strain SP15, isolated from a patient with neonatal meningitis. Thus, the expression level of luminescence of SP15(pMT3a) reflects P*clbB* activity. Relative luminescence units (RLUs) and optical density at 600 nm (OD_600_) of SP15(pMT3a) were monitored for 8 h in Dulbecco’s modified Eagle’s medium (DMEM)-HEPES at 37°C (Fig. 1a). According to RLUs normalized to OD_600_ (RLU/OD_600_), the peak of P*clbB* activity was just after 4 h. Therefore, we decided to set the measurement time point at 4 h for the following experiments. First, we tested the intrinsic luminescence variability of the WT strain SP15(pMT3a) by measuring RLU/OD_600_ for 300 isolates (Fig. 1b). Relative to (versus) the median value of RLU/OD_600_ of the 300 isolates, the values of RLU/OD_600_ of individual isolates were increased between −20% and +30%; And, relative to the median value of OD_600_, the OD_600_s of individual isolates were found to be between −20% and +30%.

**TABLE 1:** E. coli strains and plasmids used in this study

Strain or plasmid	Genotype and/or characteristics^*a*^	Reference(s) or source
SP15	SP15 with mutation in *rpsL* (Str^r^), a derivative of SP15 isolated from spinal fluid of a neonate with meningitis; O18:K1 serotype; colibactin producer	2
Nissle 1917 (EcN)	EcN with mutation in *rpsL* (Str^r^), a derivative of the probiotic strain EcN; colibactin producer	35
UTI89	Archetypal uropathogenic E. coli isolated from a patient with cystitis; colibactin producer	64
NC101	Commensal murine adherent-invasive E. coli; colibactin producer; procarcinogenic in different CRC mouse models	15
SP15 Δ*clbA*	*clbA* mutant of strain SP15; Str^r^ Kan^r^	2
SP15 Δ*clbN*	*clbN* mutant of strain SP15; Str^r^ Kan^r^	2
SP15 Δ*ppk*	*ppk* mutant of strain SP15; Str^r^ Chl^r^	This study
EcN Δ*ppk*	*ppk* mutant of strain EcN; Str^r^ Chl^r^	This study
UTI89 Δ*ppk*	*ppk* mutant of strain UTI89; Chl^r^	This study
NC101 Δ*ppk*	*ppk* mutant of strain NC101; Chl^r^	This study
SP15(pMT3a)	SP15 containing pMT3a; Str^r^ Carb^r^	This study
P1D10	SP15(pMT3a) with transposon inserted into gene *ppk*; Str^r^ Carb^r^ Kan^r^	This study
SP15(pMT3)	SP15 containing pMT3; Str^r^ Kan^r^	This study
SP15 Δ*ppk*-c	SP15Δ*ppk* containing pGEN-*ppk*; Str^r^ Chl^r^ Carb^r^	This study
EcN *clbB*::*lux*	EcN harboring the *clbB* promoter reporter (*luxCDABE*) fusion on the chromosome; Kan^r^	17, 26
EcN *clbB*::*lux Δppk*	EcN *clbB*::*lux* with gene *ppk* disrupted; Kan^r^ Chl^r^	This study
TOP10	Used as host for recombinant plasmids	Thermo Fisher
pCM17	Luciferase-encoding pCM17 vector containing the *ompC* promoter upstream of the *luxCDABE* operon	65
pMT3	*clbB* promoter reporter fusion; Kan^r^	This study
pMT3a	*clbB* promoter reporter fusion; Carb^r^	This study
pGEN-MCS	Vector of gene *ppk* for complementation of Δ*ppk*; Carb^r^	Gift from Ganwu Li
pGEN-*ppk*	pGEN-MCS bearing *ppk* sequence; Carb^r^	This study

a Str^r^, streptomycin resistance; Kan^r^, kanamycin resistance; Chl^r^, chloramphenicol resistance; Carb^r^, carbenicillin resistance.

10.1128/mSphere.01195-20.1 FIG S1 Schematic representation of P*clbB* reporter fusion. (a) The DNA sequence MT3 (490 bp) containing the promoter of *clbB* (P*clbB*) was amplified and used to be fused preceding the operon *luxABCDE* on a low-copy-number plasmid, resulting in the P*clbB*-*lux* reporter fusion pMT3a. (b) The construction of EcN *clbB*::*lux* as previously described (A. Wallenstein, N. Rehm, M. Brinkmann, M. Selle, et al., mSphere 5:e00591-20, 2020, https://doi.org/10.1128/mSphere.00591-20; S. Homburg, E. Oswald, J. Hacker, and U. Dobrindt, FEMS Microbiol Lett 275:255–262, 2007, https://doi.org/10.1111/j.1574-6968.2007.00889.x). The operon *luxABCDE* fused with a kanamycin resistance cassette was directly inserted into the bacterial chromosome preceding the start codon of *clbB*. Download FIG S1, EPS file, 0.7 MB. Copyright © 2020 Tang-Fichaux et al. 2020 Tang-Fichaux et al. This content is distributed under the terms of the Creative Commons Attribution 4.0 International license.

Next, a transposon (Tn) mutant library of SP15(pMT3a) was constructed by using the EZ-Tn5 <KAN-2>Tnp Transposome. Under the same condition as described above, RLU/OD_600_ values of 823 Tn mutants were measured at 4 h. Seventeen mutants showing growth retardation compared with the WT (increase of OD_600_ of less than −20%) were excluded; 41 mutants showed an increase of RLU/OD_600_ of less than −20% (Fig. 1c). By sequencing, 40 mutants were identified to have the Tn inserted into the *lux* operon, and 1 mutant (named P1D10) was identified to have the Tn inserted into the gene *ppk* (1,754 bp after the start codon) (Fig. S2). The attenuated P*clbB* activity in P1D10 was also observed by time course monitoring (Fig. 1d).

10.1128/mSphere.01195-20.2 FIG S2 The transposon insertion site of P1D10 is in the gene *ppk*. In P1D10, the transposon EZ-Tn5 <KAN-2> is inserted into the gene *ppk* on the chromosome, 1,754 bp after the start codon of *ppk*. *ppk* is present in an operon harboring *ppk* and *ppx*, which share the same promoter. Download FIG S2, EPS file, 0.6 MB. Copyright © 2020 Tang-Fichaux et al. 2020 Tang-Fichaux et al. This content is distributed under the terms of the Creative Commons Attribution 4.0 International license.

**FIG 2:**
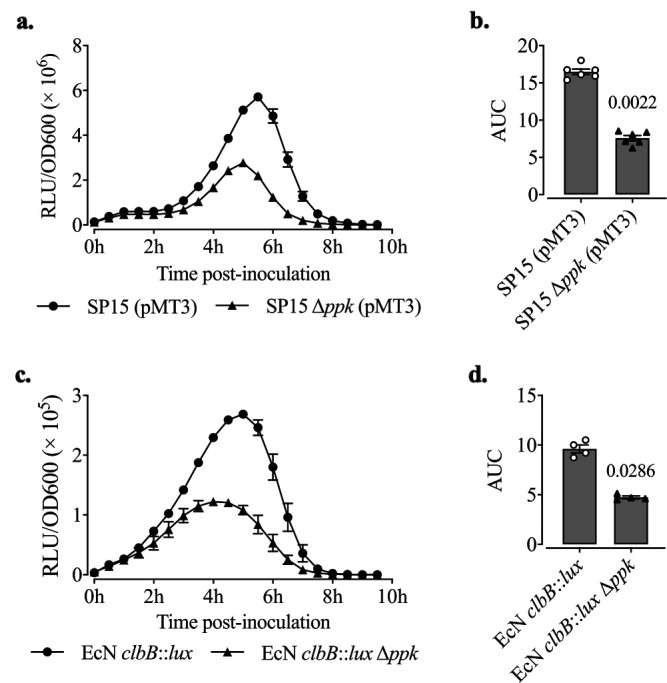
Deletion of *ppk* reduced P*clbB* activity in different genetic contexts using plasmid or chromosomal fusions. (a) Time course RLU/OD_600_ of SP15(pMT3) and SP15 Δ*ppk*(pMT3) grown in DMEM-HEPES at 37°C. (b) Areas under the curves (AUC) of RLU/OD_600_ from panel a. Bars represent means ± SEMs (*n* = 6 biological replicates). The significance was determined using the Mann-Whitney test. *P* value = 0.0022. (c) Time course RLU/OD_600_ of EcN P*clbB*-*lux* and EcN P*clbB*-*lux* Δ*ppk* grown in DMEM-HEPES at 37°C for 10 h. (d) AUC of RLU/OD600 from panel c. Bars represent means ± SEMs (*n* = 4 biological replicates). The significance was determined using the Mann-Whitney test. *P* value = 0.0286.

To confirm that the attenuated P*clbB* activity was due to the inactivation of *ppk* in P1D10, we constructed an isogenic *ppk* deletion mutant of SP15 (SP15 Δ*ppk*). SP15 Δ*ppk* carrying the P*clbB*-*lux* reporter fusion pMT3 (Table 1) had significantly lower P*clbB* activity than the WT (Fig. 2a and b). Additionally, we deleted *ppk* in the previously described reporter strain Nissle 1917 (EcN) carrying a transcriptional fusion of P*clbB* and the *lux* operon on the chromosome (EcN *clbB*::*lux*) (17, 26), resulting in strain EcN *clbB*::*lux* Δ*ppk* (Fig. S1b). P*clbB* activity was also significantly lower in EcN *clbB*::*lux* Δ*ppk* than in EcN *clbB*::*lux* (Fig. 2c and d). These results consistently suggest that PPK is an enhancer of P*clbB* activity.

### PPK is required for full production of colibactin.

**FIG 3:**
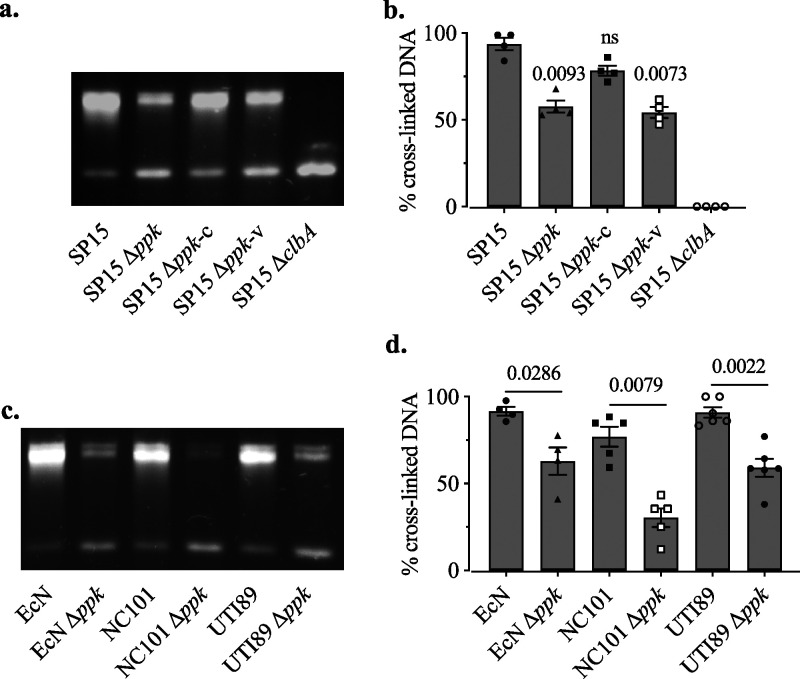
Deletion of *ppk*-reduced ICL amount induced by colibactin-producing E. coli. (a) DNA interstrand cross-link (ICL) formation caused by SP15, SP15 Δ*ppk*, the complemented strain SP15 Δ*ppk* carrying plasmid pGEN-*ppk* expressing *ppk* (SP15 Δ*ppk*-c), the control SP15 Δ*ppk* carrying the vector pGEN-MCS (SP15 Δ*ppk*-v), and the negative-control *clbA-*deletion mutant (SP15 Δ*clbA*). The DNA with ICL is nondenaturable and displays delayed migration (upper band) compared to the unaffected denatured single-stranded DNA (lower band). This image is representative of those from four independent experiments. (b) The percentage of the cross-linked DNA signal in the upper band relative to the total DNA signal in the lane was determined by image analysis. Bars represent means ± SEMs (*n* = 4 biological replicates). The significance of the difference between each strain and the WT was determined using the Kruskal-Wallis test followed by the two-stage step-up method of Benjamini, Krieger, and Yekutieli; *P* values are shown. ns, no significant difference. (c) ICL activity of WT strains EcN, NC101, and UTI89 and their respective *ppk* deletion mutants EcN Δ*ppk*, NC101 Δ*ppk*, and UTI89 Δ*ppk*. This image is representative of those from four independent experiments. (d) Percentage of cross-linked DNA signal relative to the total DNA signal in panel c. Bars represent means ± SEMs (*n* = 4 to 6 biological replicates). The significance of the difference between the Δ*ppk* mutant and the WT was determined using the Mann-Whitney test; *P* values are shown.

To determine whether PPK is associated with the biosynthesis of colibactin, we performed DNA interstrand cross-linking (ICL) assays in which bacteria were in direct contact with DNA. The ICL amount is directly correlated with the production of active colibactin (27). After incubation with bacteria, exposed DNA was purified and migrated under the alkaline-denaturing conditions. DNA with ICL is nondenaturable and displays delayed migration compared to that of unaffected denatured single-stranded DNA. Our results showed that SP15 Δ*ppk* caused less ICL than the WT, and this ability was restored in the complemented strain, SP15 Δ*ppk* carrying a plasmid, pGEN-*ppk*, expressing *ppk* (SP15 Δ*ppk*-c) (Fig. 3a and b). This *ppk* deletion-associated phenotype was also observed in other strains, including the probiotic strain EcN, the colitogenic strain NC101, and the uropathogenic strain UTI89 (Fig. 3c and d). These results indicate that PPK is required for colibactin biosynthesis in different genetic contexts.

**FIG 4:**
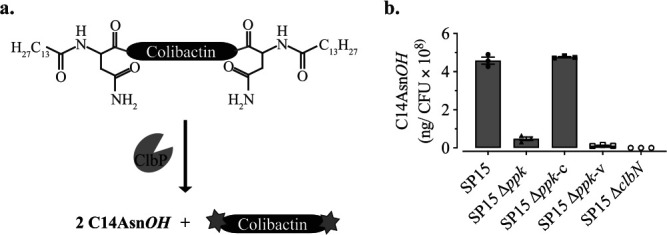
Deletion of *ppk* reduced the production of colibactin. (a) ClbP cleaves the amide bonds of precolibactin to release the prodrug motif C_14_AsnOH and active colibactin (3). (b) The amount of C_14_AsnOH produced by SP15 and the derivatives was quantified by liquid chromatography-mass spectrometry (LC-MS). Bacteria were cultivated in DMEM-HEPES at 37°C for 8 h, and C_14_AsnOH in the supernatant was quantified. The results were normalized to CFU and are presented as the quantity of C_14_AsnOH. The bars represent means ± SEMs (*n* = 3 biological replicates).

Since colibactin cannot be directly quantified yet, we quantified the production of the prodrug motif C_14_AsnOH, which is correlated with colibactin production and maturation (Fig. 4a) (3). The result showed that the production level of C_14_AsnOH of SP15 Δ*ppk* was about 10 times less than that of the WT (Fig. 4b), and this level was restored to the WT level in SP15 Δ*ppk*-c. Taken together, these findings indicate that PPK is required for full P*clbB* activity, thereby enhancing colibactin biosynthesis.

### PPK is required for full genotoxicity of colibactin-producing E. coli.

**FIG 5:**
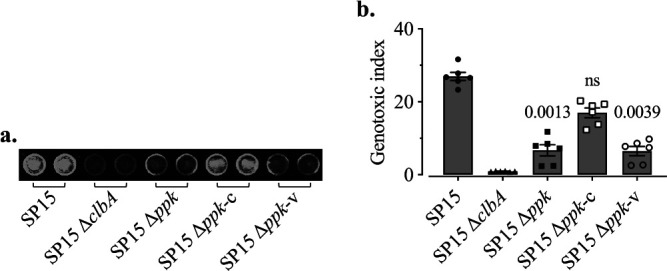
Deletion of *ppk* reduces the genotoxicity of colibactin-producing E. coli in eukaryotic cells. (a) HeLa cells after a transient infection with SP15, SP15 Δ*ppk*, SP15 Δ*ppk*-c, SP15 Δ*ppk*-v, or SP15 Δ*clbA*. The multiplicity of infection (MOI) was 100. The signal of γH2AX is pseudocolored in green and the signal of DNA in red. This image is representative of those from three independent experiments performed with two independent bacterial cultures. (b) The genotoxic index was determined by quantification of the signal of γH2AX relative to DNA content and normalized to the control without infection. Bars represent means ± SEMs (*n* = 6 independent experimental replicates). The significance of the difference between each strain and WT was determined using the Kruskal-Wallis test followed by the two-stage step-up method of Benjamini, Krieger, and Yekutieli; *P* values are shown.

As ICLs induce the DNA damage response in the host cells, we quantified γH2AX, which is a sensitive marker for colibactin-induced DNA damage by in-cell Western (ICW) assay (2). After a 4-h transient infection and 4 h of growth, HeLa cells grown on a 96-well plate were fixed, and γH2AX was stained by immunofluorescence. The fluorescent signal of γH2AX is pseudocolored in green, and the fluorescent signal of DNA is pseudocolored in red (Fig. 5a). The genotoxic index was determined by quantification of the signal of γH2AX relative to DNA content and normalized to the control (Fig. 5b). The results showed that the genotoxicity of SP15 Δ*ppk* was significantly lower than that of the SP15 WT and was restored in SP15 Δ*ppk*-c. This indicates that PPK is required for the full genotoxicity of SP15.

**FIG 6:**
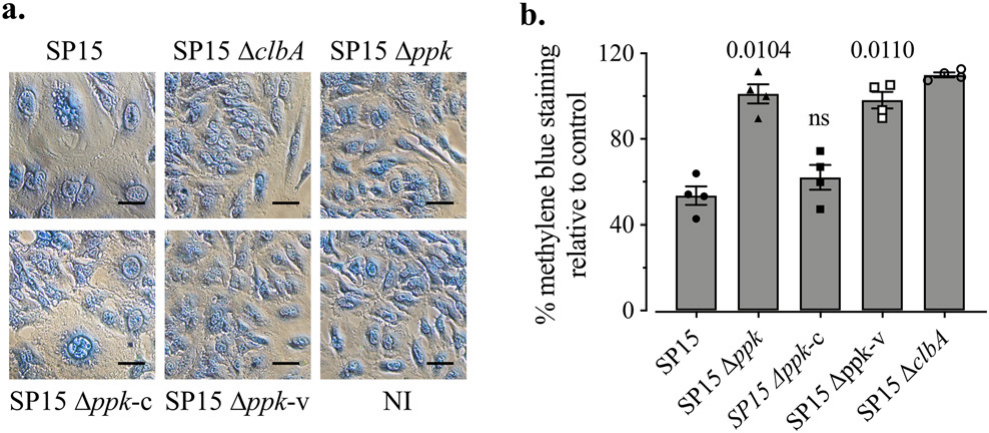
Deletion of *ppk* abolished the ability of colibactin-producing E. coli to induce megalocytosis of eukaryotic cells. (a) HeLa cells stained with methylene blue after a transient infection with SP15 Δ*ppk*-c, SP15 Δ*ppk*-v, or SP15 Δ*clbA* at an MOI of 100. NI, not infected. The scale bars represent 50 μm. After a 4-h infection, HeLa cells were washed and incubated for 72 h in the presence of gentamicin. Then, the cells were fixed and stained with methylene blue. This image is representative of those from 4 independent experiments. (b) The cell viability relative to that of NI controls was determined by quantification of methylene blue staining. The methylene blue was extracted and quantified by the measurement of OD_660_. Bars represent means ± SEMs (*n* = 4 independent experimental replicates). The significance of the difference between each strain and the WT was determined using the Kruskal-Wallis test followed by the two-stage step-up method of Benjamini, Krieger, and Yekutieli; *P* values are shown.

Colibactin-producing E. coli induces megalocytosis in cultured eukaryotic cells, characterized by a progressive enlargement of the cell body and nucleus and a reduced cell number (1). To corroborate the previous result, we investigated the role of PPK in megalocytosis. Fewer giant cells were observed with infection by SP15 Δ*ppk* than for cells infected by the WT (Fig. 6a). Through the quantification of stained methylene blue on infected HeLa cells relative to noninfected cells, our results indicate that the mutation of *ppk* significantly reduced the ability of SP15 to induce megalocytosis, which was restored in SP15 Δ*ppk*-c (Fig. 6b). This indicates that PPK is required for colibactin-producing E. coli to induce DNA damage and subsequent megalocytosis of host cells.

### Mesalamine reduces P*clbB* activity and represses colibactin production.

**FIG 7:**
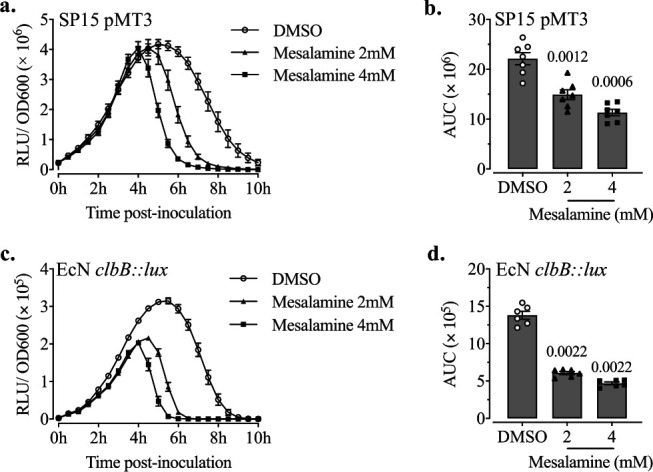
Mesalamine treatment reduces P*clbB* activity. (a) Time course RLU/OD_600_ of SP15(pMT3) with the treatment of mesalamine (2 mM and 4 mM) and the solvent DMSO as a control in DMEM-HEPES at 37°C. (b) AUC of RLU/OD_600_ from panel a. The symbol and bars represent means ± SEMs (*n* = 7 biological replicates). The significance compared with the control (DMSO) was determined using the Mann-Whitney test; the *P* value is shown. (c) RLU/OD_600_ of EcN *clbB*::*lux* with treatment and growing conditions as before. (d) AUC of RLU/OD_600_ from panel c. The symbol and bars represent means ± SEMs (*n* = 6 biological replicates). The significance compared with the control (DMSO) was determined using the Mann-Whitney test; the *P* value is shown.

One well-known PPK enzymatic activity inhibitor is the anti-inflammatory drug mesalamine, commonly prescribed for IBD and proposed for CRC prevention (25). We thus investigated whether mesalamine has an effect on colibactin synthesis similar to what we observed in Δ*ppk* mutants. First, we tested the effect of mesalamine on P*clbB* activity in two genetic backgrounds, SP15 and EcN. Luminescence emitted by the bacteria was monitored in DMEM-HEPES at 37°C with or without the presence of mesalamine (2 or 4 mM). The results showed that mesalamine reduced P*clbB* activity in a dose-dependent manner in both SP15 (Fig. 7a and b) and EcN (Fig. 7c and d), while it did not cause growth retardation of bacteria (Fig. S3). This indicates that mesalamine has an inhibitory effect on P*clbB* activity.

10.1128/mSphere.01195-20.3 FIG S3 Mesalamine does not reduce the growth rate of SP15 and EcN. (a) Growth of SP15(pMT3) with treatment with mesalamine (2 mM and 4 mM) and the solvent DMSO in DMEM-HEPES at 37°C. The symbol and bar represent mean ± SEM (*n* = 7 biological replicates). (b) Growth of EcN *clbB*::*lux* with treatment and the growing conditions as before. The symbol and bars represent means ± SEMs (*n* = 6 biological replicates). Download FIG S3, EPS file, 0.1 MB. Copyright © 2020 Tang-Fichaux et al. 2020 Tang-Fichaux et al. This content is distributed under the terms of the Creative Commons Attribution 4.0 International license.

**FIG 8:**
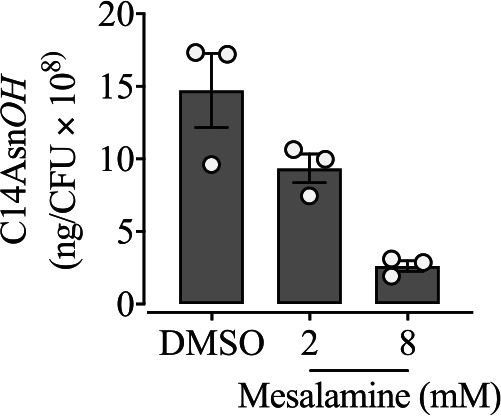
Mesalamine represses colibactin production. Shown is quantification of C_14_AsnOH produced by SP15 with treatment with mesalamine (2 mM and 8 mM) and the solvent DMSO. Bacteria were cultivated in DMEM-HEPES (in the presence of mesalamine or DMSO) at 37°C for 8 h, and C_14_AsnOH in the supernatant was quantified. The results were normalized to CFU and are presented as the quantity of C_14_AsnOH. The bars represent means ± SEMs (*n* = 3 biological replicates).

To test whether mesalamine treatment has an impact on the production of colibactin, we quantified the production of the prodrug motif C_14_AsnOH of the bacteria with or without mesalamine. We observed that a dose of 8 mM mesalamine decreased about 6 times the C_14_AsnOH level (Fig. 8) without affecting bacterial viability (Fig. S4). This result indicates that mesalamine inhibits the biosynthesis of colibactin.

10.1128/mSphere.01195-20.4 FIG S4 Mesalamine treatment does not reduce the viability of SP15. Shown are CFU per milliliter after 8 h of culturing in DMEM-HEPES (in the presence of mesalamine or DMSO) at 37°C. The quantification of C_14_AsnOH is shown in Fig. 8 in the main text. The bars represent means ± SEMs (*n* = 3 biological replicates). Download FIG S4, EPS file, 0.1 MB. Copyright © 2020 Tang-Fichaux et al. 2020 Tang-Fichaux et al. This content is distributed under the terms of the Creative Commons Attribution 4.0 International license.

**FIG 9:**
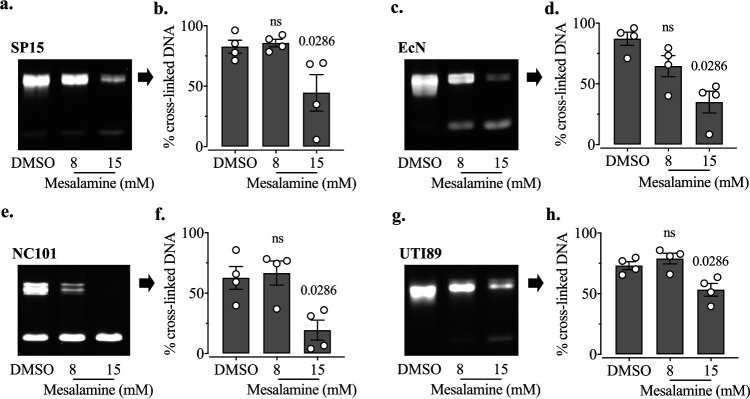
Mesalamine inhibits ICL activity of colibactin-producing E. coli. The strains SP15 (a and b), EcN (c and d), NC101 (e and f), and UTI89 (g and h) were inoculated at 1.5 × 10^6^ CFU in 100 μl of DMEM-HEPES, with treatment with mesalamine (8 mM and 15 mM), and the solvent DMSO (control, 4% end concentration, similar to the end concentration of DMSO in samples treated with 15 mM mesalamine). After 4 h of incubation at 37°C, bacteria were spun down and resuspended with sterile Milli-Q H_2_O. Then, 500 ng of linearized pUC19 plasmid was added into each resuspension. After 40 min of incubation at 37°C, DNA was purified, loaded onto an agarose gel, and migrated under alkaline denaturing conditions. DNA with covalent ICLs is nondenaturable and displays delayed migration compared to denatured single-stranded DNA (lower band). The percentage of the DNA signal in the upper (cross-linked DNA band) relative to the total DNA signal in the lane was determined by image analysis. (c, e, and g) The photos are representative of those from four experiments. (d, f, and h) The quantifications of cross-linked DNA were determined as previously described. The bars represent means ± SEMs (*n* = 4 independent experimental replicates). The significance compared with the control (DMSO) was determined using the Mann-Whitney test; the *P* value is shown.

**FIG 10:**
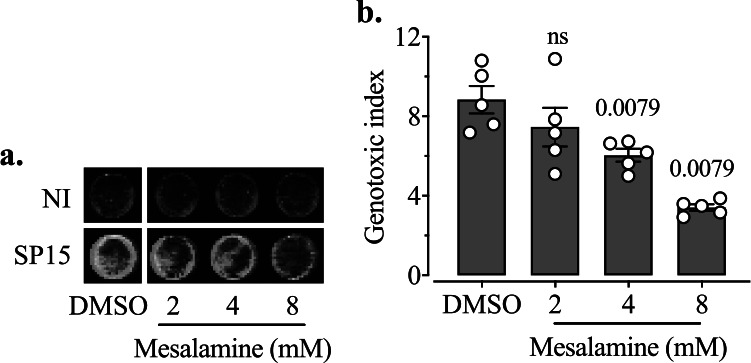
Mesalamine inhibits the genotoxicity of colibactin-producing E. coli. (a) HeLa cells after a transient infection with SP15 under the treatment with mesalamine (2 mM, 4 mM, or 8 mM) and the solvent DMSO (control). The MOI was 100. The signal of γH2AX is green, and the signal of DNA is red. This image is representative of those from four independent experiments. (b) The genotoxic index was determined by quantification of the signal of γH2AX relative to DNA content and normalized to the control without infection. The bars represent means ± SEMs (*n* = 5 independent experimental replicates). The significance of the difference between each strain and the control was determined using the Kruskal-Wallis test followed by the two-stage step-up method of Benjamini, Krieger, and Yekutieli; *P* values of <0.05 are shown.

We investigated the effect of mesalamine on ICL formation induced by various colibactin-producing E. coli strains, including SP15 (Fig. 9a and b), EcN (Fig. 9c and d), NC101 (Fig. 9e and f), and UTI89 (Fig. 9g and h). We observed that a dose of 15 mM mesalamine significantly reduced ICL formation in all the strains tested, while the bacterial CFU were not reduced and even showed a slight increase with the treatment with mesalamine (Fig. S5). To confirm these results, we also evaluated the genotoxicity in eukaryotic cells induced by colibactin-producing E. coli with or without mesalamine treatment. By using ICW assays, we observed that the genotoxicity induced by SP15 was reduced in a dose-dependent manner with the treatment of mesalamine (Fig. 10), while the viability of HeLa cells was not affected (Fig. S6a), and the bacterial CFU were not reduced, by mesalamine (Fig. S6b). Taken together, these results demonstrated that treatment with mesalamine inhibited the production of colibactin, thereby protecting eukaryotic cells from the genotoxicity of colibactin-producing E. coli.

10.1128/mSphere.01195-20.5 FIG S5 Mesalamine does not reduce the viability of E. coli. In ICL experiments (data shown in Fig. 9 in the main text), CFU per milliliter of SP15 (a), EcN (b), NC101 (c), and UTI89 (d) were determined after 4 h of growth in DMEM-HEPES with treatment with mesalamine (8 mM and 15 mM) or the solvent DMSO. The bars represent means ± SEMs (*n* = 4 independent experimental replicates). The significance compared with the control (DMSO) was determined using the Mann-Whitney test. The *P* value is shown. ns, no significance. Download FIG S5, EPS file, 0.6 MB. Copyright © 2020 Tang-Fichaux et al. 2020 Tang-Fichaux et al. This content is distributed under the terms of the Creative Commons Attribution 4.0 International license.

10.1128/mSphere.01195-20.6 FIG S6 Mesalamine treatment did not affect the viability of HeLa cells and did not cause growth retardation of the E. coli strain SP15. (a) The percentage of HeLa cell viability was determined by comparing the DNA content with the treatment mesalamine/DMSO to the control without treatment (NT) in the ICW assays (presented in Fig. 10 in the main text). The bars represent means ± SEMs (*n* = 4 independent experimental replicates). The significance of the difference between HeLa cells treated with mesalamine and DMSO was not found by using the Mann-Whitney test. (b) CFU per milliliter after 4 h of infection. The bars represent means ± SEMs (*n* = 5 independent experimental replicates). The significance of the difference between bacteria treated with mesalamine and DMSO was determined by using the Mann-Whitney test; *P* values of <0.05 are shown. Download FIG S6, EPS file, 0.1 MB. Copyright © 2020 Tang-Fichaux et al. 2020 Tang-Fichaux et al. This content is distributed under the terms of the Creative Commons Attribution 4.0 International license.

We then investigated the impact of mesalamine treatment in a Δ*ppk* mutant on P*clbB* activity, colibactin production, and genotoxicity. We observed that mesalamine decreased P*clbB* activity (Fig. S7a and b), the production level of C_14_AsnOH (Fig. S7d), and genotoxicity (Fig. S7f and g), without reducing bacterial viability (Fig. S7c, e, and h). These results indicate that mesalamine inhibits P*clbB* activity and colibactin production independently from its inhibition effect on PPK enzymatic activity (25).

10.1128/mSphere.01195-20.7 FIG S7 Mesalamine also inhibits P*clbB* activity and colibactin production independently from PPK. In SP15 Δ*ppk*, with treatment with mesalamine and the solvent DMSO (control), P*clbB* activity (a and b) and growth (c) were monitored for 10 h, the production level of C_14_AsnOH (d) and CFU per milliliter (e) were quantified, and the ICL activity was tested (f and g) and CFU per milliliter were quantified (h). The symbol and bars represent means ± SEMs (*n* = 3 or 5 biological replicates). In panels g and h, the significance compared with the control (DMSO) was determined using the Mann-Whitney test; the *P* value is shown. Download FIG S7, EPS file, 2.1 MB. Copyright © 2020 Tang-Fichaux et al. 2020 Tang-Fichaux et al. This content is distributed under the terms of the Creative Commons Attribution 4.0 International license.

## DISCUSSION

The data implicating colibactin in virulence and colorectal tumorigenesis have motivated extensive structural and pharmacological studies of colibactin (5, 28–33) and other metabolites of the *pks* pathway (2, 34, 35). However, very limited data are available about the regulation of the production of this important genotoxin. In this study, we developed a high-throughput screening of regulators involved in colibactin biosynthesis based on the construction of a library of random mutants of a colibactin-producing E. coli strain harboring a P*clbB*-reporter fusion. We reasoned that because ClbB is essential for the production of colibactin, regulators of P*clbB* activity should impact the production level of colibactin. Of 823 mutants screened, 1 mutant had lower P*clbB* activity than the WT. This mutant had the transposon inserted in the gene *ppk*, encoding the polyphosphate kinase (PPK). We then constructed isogenic mutants of *ppk* in different colibactin-producing E. coli strains to test P*clbB* activity and the production of colibactin as well. Consistently, the deletion of *ppk* reduced P*clbB* activity and caused a lower production level of colibactin.

This work highlights the role of PPK in P*clbB* activity, which is correlated with the production of colibactin. A recent study has shown that ClbR is the transcriptional activator of *clbB* (17). In this work, we discovered the first regulator of *clbB* transcriptional activity outside of the *pks* island. PPK is essential for the production of long-chain polyP (36). E. coli mutants lacking *ppk* were described to be defective in virulence and responses to multiple stresses (i.e., nutrient starvation, oxidants, acidic challenge, osmotic shock, and heat shock) (24, 37, 38). Additionally, the *ppk* deletion mutant of meningitis E. coli strain E44 showed less ability than the WT to cross the blood-brain barrier (BBB) (37). The *ppk* deletion mutant of the uropathogenic strain UTI89 was shown to have defects in biofilm formation, resistance to oxidation, and formation of antibiotic-resistant persister cells (25, 39). PPK is distributed across a wide spectrum of bacterial pathogens and absent in mammalian cells, and it has been therefore proposed as a new target for developing antibacterial agents that specifically target pathogens without affecting the host and its beneficial bacteria (40). In this work, we observed the deletion of *ppk* reduced the genotoxicity of colibactin-producing E. coli, including the meningitic strain SP15, the probiotic strain EcN, the colitogenic strain NC101, and the uropathogenic strain UTI89. Future research should clarify whether this is the case *in vivo*. Our finding reinforces the idea to take PPK as a target of antibacterial drugs and provided a new path for developing an anticolibactin strategy.

Several studies have focused on finding inhibitors of PPK (25, 41–43). One of the identified PPK inhibitors, mesalamine (also known as mesalazine or 5-aminosalicylic acid), has been validated by treating different bacteria ranging from clinically isolated uropathogenic E. coli and P. aeruginosa strains to human gastrointestinal luminal samples (25). Mesalamine is a drug commonly used to treat IBD patients, and rare side effects have been reported (44–46). Mesalamine exerts its anti-inflammatory effects locally on the colorectal mucosa, and the efficacy is dependent on achieving high intraluminal concentrations (47, 48). In patients conventionally treated with mesalamine, stool concentrations of mesalamine are on the median order of 30 mM, ranging from 10 to 100 mM; these concentrations correspond to luminal concentrations of mesalamine 100 times greater than the concentrations in the colonic mucosa (49). Mesalamine has been shown to have chemopreventive effects on CRC and has been proposed as a first-line treatment that should be given daily in high doses and long term to reduce the possibility of recurrence and risk of CRC (45, 50, 51). The effects of mesalamine on the host have been intensely researched (51–56), while few studies have investigated the effects on bacteria. Mesalamine has been shown to affect bacterial gene expression (49) and to alter gut microbiota (57–59). Interestingly, a recent report showed that mesalamine downregulated the transcription of the *pks* gene (60), but it did not show which *pks* gene was downregulated. This study also showed that mesalamine (9.8 mM and 13 mM) inhibited DNA breakage in colonic epithelial Caco-2 cells induced by colibactin-producing E. coli (60). In our study, we first identified PPK as an enhancer of colibactin production, which led us to test the PPK inhibitor mesalamine. We tested not only the inhibitory effects of mesalamine on the genotoxicity of colibactin-producing E. coli in eukaryotic cells but also directly the amount of colibactin-correlated metabolite C_14_AsnOH and the formation of ICL. We also tested a wider range of colibactin-producing E. coli strains and demonstrated that the effect of mesalamine on colibactin production is universal. Among the strains tested, one strain should especially get our attention: the probiotic strain EcN, which is the active component of microbial drug Mutaflor (61). EcN has been widely used in the treatment of IBD and has proven to be as effective as the gold standard mesalamine for the maintenance of remission in ulcerative colitis patients (61). It has been suggested that a combination of mesalamine and EcN might exert additive or synergistic therapeutic efficacy, and mesalamine has no effect on the viability of EcN *in vivo* (62). Here, our data suggest that *in vitro* mesalamine has a suppressive effect on the genotoxicity of EcN without altering the viability of EcN. Future research should clarify whether this is the case *in vivo*.

In this study, we also investigated whether mesalamine treatment inhibits the biosynthesis of colibactin in a Δ*ppk* mutant. Interestingly, an additional inhibition effect on colibactin production was observed in the Δ*ppk* mutant treated with mesalamine, indicating that mesalamine is capable of inhibiting colibactin production independently from its inhibitory effect on PPK enzymatic activity (25). Future research is needed to clarify this new mechanism.

In summary, this study showed that PPK played a role in the transcriptional activity of *clbB* and was required for the genotoxicity of colibactin-producing E. coli. This provided us a new perspective on the regulatory network of colibactin production and brought us a novel clue for anticolibactin strategy development. By using the PPK inhibitor mesalamine, we confirmed the role of PPK in colibactin production and also identified mesalamine as an effective drug for inhibiting *pks^+^* E. coli genotoxicity to eukaryotic cells. Further studies are necessary to test the synergistic activity of mesalamine and EcN *in vivo* and to determine if treatment of IBD with both mesalamine and EcN protects patients against CRC.

## MATERIALS AND METHODS

### Bacterial strains, plasmids, and growth conditions.

The bacterial strains and plasmids used in this study are listed in Table 1. Gene mutagenesis was performed by using the λ red mutagenesis method with the primers listed in Table 2 and confirmed by PCR. For genetic manipulations, all E. coli strains were grown routinely in lysogenic broth (LB) medium. When appropriate, antibiotics were added at the following concentrations: 50 μg/ml for kanamycin, 50 μg/ml for carbenicillin, and 25 μg/ml for chloramphenicol.

**TABLE 2:** Primers used in this study

Primer	Sequence (5′–3′)^*a*^	Aim
MT3_P*clbB*-EcoRI-F	CCGGAATTCCTTTGAACTTATCCATGTTTCC	Cloning of DNA sequence MT3 containing the promoter of *clbB*
MT1_P*clbB*-BamHI-R	CGCGGATCCCAGAGGTATTATCCATAACCATCAC	
MT43_*ppk*-mut-F	CGCCATAATATCCAGGCAGTGTCCCGTGAATAAAACGGAGTAAAAGTGGTA**TGTGTAGGCTGGAGCTGCTTCG**	Deletion of *ppk*
MT44_*ppk*-mut-R	GTTATTCAGATTGTTCGAGTGATTTGATGTAGTCGTAAATCGCCAACTGCG**CATATGAATATCCTCCTTAGTTC**	
MT54_pGEN-*ppk*-HindIII-F	CCGAAGCTTGTACATCGGTGCATTTCGTC	Amplification of *ppk* plus its putative promoter region
MT55_pGEN-*ppk*-BamHI-R	CGCGGATCCAGGGTTATTCAGATTGTTCGAG	

a Restriction enzyme sites are underlined, and priming sites for amplifying resistance gene are written in bold.

### Chemicals and reagents.

Unless otherwise indicated, chemicals were from Sigma-Aldrich or Fisher. The stock solution of mesalamine (400 mM) was extemporaneously prepared in dimethyl sulfoxide (DMSO), and dilutions were made immediately before each experiment.

### Plasmid construction.

The plasmids used in this study are listed in Table 1. For the construction of *clbB* promoter (P*clbB*) reporter fusions, the promoter sequence of *clbB* (from bp −473 to +17 relative to the initiation start codon of *clbB*), 490 bp, named MT3, containing P*clbB* was amplified from the genome of SP15 and cloned into the reporter plasmid pCM17 preceding the *luxCDABE* operon (Fig. S1). The primers used are listed in Table 2. The result plasmid, pMT3, was verified by sequencing. After pMT3 is introduced into the target bacteria, the *luxCDABE* operon encodes a luciferase (LuxA and LuxB) and the enzymes that produce its substrate (LuxC, LuxD, and LuxE) under the control of P*clbB*, so bacteria that have P*clbB* activated and express the cluster emit 490-nm luminescence spontaneously. The promoter-reporter fusion pMT3 contains the kanamycin resistance (Kan^r^) cassette. For compatibility with the transposon containing the Kan^r^ cassette, the Kan^r^ cassette of pMT3 was disrupted by inserting an ampicillin resistance (Amp^r^) gene at the restriction site BssHII, which resulted in pMT3a. The plasmids were verified by sequencing. For complementation, the coding sequence of gene *ppk* plus its putative promoter region was amplified (the primers used are listed in Table 2) and cloned into pGEN-MCS using HindIII and BamHI restriction sites. All restriction enzymes were purchased from New England BioLabs (NEB) and used based on the supplier’s recommendations.

### Construction of Tn mutant library and identification of Tn insertion sites of selected mutants.

The transposon (Tn) mutant library of E. coli strain SP15 containing pMT3a was prepared using the EZ-Tn5 <KAN-2>Tnp Transposome kit (Lucigen). Mutants were stored at –80°C with 20% (vol/vol) glycerol as a cryoprotectant. To identify Tn insertion sites of selected mutants, DNA fragments spanning the Tn insertion junction were amplified by arbitrarily primed PCR (AP-PCR) for sequence analysis (63), and then the resulting sequence was mapped to the bacterial genome and plasmids.

### Luminescence measurement.

For monitoring *clbB* promoter (P*clbB*) activity in SP15 (carrying pMT3a or pMT3) and the mutants, each strain was inoculated into 150 μl of LB and grown at 37°C without shaking. A total of 5 μl of overnight culture was inoculated into 100 μl of Dulbecco’s modified Eagle’s medium (DMEM)-HEPES (Gibco) in a black 96-well plate (Greiner Bio-One), and then the bacteria were grown without shaking at 37°C. The luminescence emission (relative light units [RLU]; 2,000-ms aperture per sample) and the optical density at 600 nm (OD_600_) were measured at 4 h by a luminometer (Tecan Spark multimode reader). To have the time course P*clbB* activity, the bacteria were grown without shaking at 37°C in the luminometer, and RLU and OD_600_ were measured every 0.5 h. The area under the curve (AUC) of RLU/OD_600_, which quantifies the cumulative luminescence, was calculated with GraphPad Prism (version 8.0) software.

To monitor P*clbB* activity in EcN *clbB*::*lux* (Fig. S1b) (17, 26) and the derivatives, each strain was inoculated into 3 ml of LB and grown at 37°C with shaking at 240 rpm overnight. A total of 500 μl of overnight culture was inoculated into 9.5 ml of DMEM-HEPES and then grown at 37°C with shaking at 240 rpm for 8h. Ten-microliter subcultures were inoculated into 100 μl of DMEM-HEPES in a black 96-well plate. Bacteria were grown without shaking at 37°C in the luminometer, and RLU and OD_600_ were measured at 0.5 h. The AUC was determined as previously described. To detect the effect of mesalamine on P*clbB* activity in EcN and SP15, the same protocol was used; mesalamine was added in 100 μl of DMEM-HEPES in the black 96-well plate inoculated with 10-μl subcultures.

### C_14_AsnOH (colibactin cleavage product) quantification.

Each E. coli strain was inoculated in triplicate into 3 ml of LB and grown at 37°C with shaking at 240 rpm overnight. A total of 500 μl of overnight culture was inoculated into 9.5 ml of DMEM-HEPES and then grown at 37°C with shaking at 240 rpm to an optical density at OD_600_ of 0.4 to ∼0.6. Then 500 μl of subculture was inoculated into 9.5 ml of DMEM-HEPES and grown under the same condition for 8 h. Bacterial cells were pelleted by centrifugation at 5,000 × *g* for 10 min, and the supernatants were filtered through a 0.22-μm-pore-size polyvinylidene difluoride (PVDF) filter (Millipore). The supernatants were stored at –80°C until *N*-myristoyl-d-asparagine (C_14_AsnOH) extraction. With the same protocol for lipid extraction as previously described (34), 5 μl of internal standard (IS) mixture (deuterium-labeled compounds) (400 ng/ml) and 0.3 ml of cold methanol (MeOH) was added to each 1-ml supernatant sample. An Oasis HLB 96-well plate was conditioned with 500 μl of MeOH and 500 μl of 10% MeOH/H_2_O. The samples were loaded in this conditioned plate and then washed with 500 μl of 10% MeOH/H_2_O and dried under aspiration. Lipids were eluted with 750 μl of MeOH, evaporated twice under N_2_, and then suspended in 10 μl of methanol. The quantification of C_14_AsnOH was performed by the MetaToul Lipidomics Facility (Inserm UMR1048, Toulouse, France), using an in-house quantification assay by high-performance liquid chromatography/tandem mass spectrometry analysis.

### Genotoxicity assay.

HeLa cells (1.5 × 10^5^/200 μl/well) were grown in DMEM GlutaMAX supplemented with 10% fetal calf serum (FCS) and 1% nonessential amino acids (NEAA), in 96-well culture plates, at 37°C in a 5% CO_2_ incubator for 24 h. Each E. coli strain was inoculated into 3 ml of LB and grown at 37°C with shaking at 240 rpm overnight. A total of 500 μl of overnight culture was inoculated into 9.5 ml of DMEM-HEPES and then grown at 37°C with shaking at 240 rpm to an OD_600_ of 0.4 to ∼0.6. Then HeLa cells were infected at a multiplicity of infection (MOI) of 100, 50, 25, or 12.5 with each strain with or without mesalamine. At 4 h postinfection, the cells were washed 3 times with Hanks’ balanced salt solution (HBSS) and incubated at 37°C in DMEM GlutaMAX supplemented with FCS and NEAA for 3 h with 200 μg/ml of gentamicin. The in-cell Western (ICW) procedure was performed as previously described (2). Briefly, after cells were fixed, permeabilized, and blocked, they were incubated overnight at 4°C with rabbit monoclonal anti-γH2AX antibody 9718 (Cell Signaling Technology; 1:200). An infrared fluorescent secondary antibody absorbing at 800 nm (IRDye 800CW, 1:500; Rockland Immunochemicals) was then applied. DNA was counterstained with RedDot2 (Biotium; 1:500). DNA and γH2AX were visualized simultaneously using an Odyssey infrared imaging scanner (LI-COR Biosciences) at 680 nm and 800 nm. Relative fluorescent units for γH2AX per well (as determined by the 800-nm signal divided by the 700-nm signal) were divided by untreated controls to determine the genotoxic index.

### DNA cross-linking assay.

The assay was performed as previously described (27). Briefly, linearized DNA was obtained by digesting plasmid pUC19 with BamHI (NEB). Each E. coli strain was inoculated into 3 ml of LB and grown at 37°C with shaking at 240 rpm overnight. A total of 500 μl of overnight culture was inoculated into 9.5 ml of DMEM-HEPES and then grown at 37°C with shaking at 240 rpm to an OD_600_ of 0.4 to ∼0.6. For bacterium-DNA interactions, 1.5 × 10^6^ bacteria were inoculated into 100 μl of DMEM-HEPES with or without mesalamine for 4 h at 37°C without shaking. Following centrifugation for 10 min at 5,000 × *g*, bacteria were pelleted and resuspended in sterile Milli-Q H_2_O. Then, 500 ng of linearized DNA was added into the bacterial suspension and incubated for 40 min at 37°C without shaking. The bacteria were then pelleted by centrifugation for 5 min at 5,000 × *g*, and the DNA was extracted from the supernatant by purification using a PCR purification kit (Qiagen) according to the manufacturer’s recommendations.

A denaturing agarose gel was prepared by dissolving 1.0 g of agarose in 100 ml of a 100 mM NaCl and 2 mM EDTA solution (pH 8.0). The gel was then soaked (2 h) in an alkaline running buffer solution (40 mM NaOH and 1 mM EDTA [pH ∼12.0]). A total of 100 ng of each DNA sample was loaded onto the agarose gel. The gel was run for 45 min at 1 V/cm and then 2 h at 2 V/cm. The gel was then neutralized for a total of 45 min in a 100 mM Tris (pH 7.4) buffer solution containing 150 mM NaCl. The gel was stained with GelRed for 20 min and revealed with UV exposure using the ChemiDoc imaging system (Bio-Rad).

### Megalocytosis assay.

Quantification of the colibactin-associated genotoxic effect by megalocytosis assay was performed as previously described (1). Briefly, HeLa cells (5 × 10^3^/well) were grown in DMEM GlutaMAX (Gibco) supplemented with 10% (vol/vol) FCS (Eurobio) and 1% (vol/vol) NEAA (Invitrogen), in 96-well culture plates, at 37°C in a 5% CO_2_ incubator for 24 h. Each E. coli strain was inoculated into 3 ml of LB and grown at 37°C with shaking at 240 rpm overnight. A total of 500 μl of overnight culture was inoculated into 9.5 ml of DMEM-HEPES and then grown at 37°C with shaking at 240 rpm to an OD_600_ of 0.4 to ∼0.6. Then HeLa cells were infected at MOIs of 100 and 50 with each strain in 100 μl of DMEM-HEPES. At 4 h postinfection, the cells were washed 3 times with HBSS (Gibco) and incubated in DMEM GlutaMAX supplemented with FCS, NEAA, and 200 mg/ml of gentamicin for 72 h before fixation (4% formaldehyde) and protein staining with methylene blue (1% [wt/vol] in 0.01 M Tris-HCl). The methylene blue was extracted with 0.1 M HCl. Staining was quantified by measurement of the OD_660_.

### Statistical analyses.

The mean and the standard error of the mean (SEM) are shown in the figures, unless otherwise stated. *P* values were calculated in GraphPad Prism 8.0 by the Mann-Whitney test or Kruskal-Wallis test followed by the two-stage step-up method of Benjamini, Krieger, and Yekutieli. *P* values of <0.05 were considered statistically significant.
